# Evaluation of the safety and effectiveness of combination therapy with 1% ivermectin solution in moderate to severe rosacea: a cohort study

**DOI:** 10.1099/jmm.0.001974

**Published:** 2025-03-07

**Authors:** Kyzzhibek Keneshbek kyzy, Aisuluu Omurzakova, Venera Azhikulova, Bukatcha Zainalieva, Dinara Abdulkasymova

**Affiliations:** 1 Faculty of International Medicine, Osh State University, 331 Lenin Str, Osh, Kyrgyz Republic; 2 Medicine Faculty, Osh State University, 331 Lenin Str, Osh, Kyrgyz Republic

**Keywords:** antiparasitic drugs, clinical manifestations, demodicosis, pink acne, side effects, skin diseases

## Abstract

**Introduction.** Rosacea is a common chronic dermatological disease that negatively affects the quality of life of patients.

**Hypothesis.** Combination therapy with 1% ivermectin solution alongside systemic treatment methods is more effective in reducing the severity of moderate to severe papulopustular rosacea symptoms compared with traditional treatment methods alone.

**Aim.** This study is aimed at finding new approaches to the treatment of moderate and severe forms of rosacea.

**Methodology.** This research compared the main group receiving ivermectin with a control group getting conventional treatment to assess the safety and efficacy of 1% ivermectin combination therapy in 70 individuals with moderate to severe rosacea. At weeks 2, 4 and 8, the dynamics of clinical symptoms were evaluated using a 4-point scale.

**Results.** The use of combination therapy with ivermectin led to a more pronounced improvement in the clinical picture. Already at week 4, a 48–54% decrease in symptoms was recorded in the main group, while in the control group, it was only by 25–30%. By week 8, an almost complete reduction of the main manifestations was observed in the main group (78–88% decrease). Regression analysis confirmed that combination therapy with ivermectin was a key factor determining a more substantial clinical improvement, regardless of age, gender and the initial severity of the patient’s condition. In addition, a much more pronounced decrease in the number of Demodex ticks was recorded in the main group. The effectiveness of combination therapy with ivermectin did not depend on the demographic or clinical characteristics of patients, which makes it a universal method of treating rosacea. No serious side effects have been reported in any patient receiving combination therapy, which indicates its safety.

**Conclusion.** This approach can be a valuable addition to existing strategies for the treatment of this chronic dermatological disease.

## Introduction

Rosacea is a chronic inflammatory disease of the skin of the face, which is one of the most common dermatological problems amongst the adult population. Despite substantial progress in understanding the pathogenesis of rosacea, effective methods of its treatment are still the subject of active scientific research. Rosacea affects from 1 to 3% of the world’s population, which underlines its status as a widespread chronic dermatological disease that has a substantial impact on the quality of life and health of people in various countries [1]. According to Mukhanova *et al*. [2], as of 2022, skin diseases are one of the substantial public health problems, accounting for 4.7% of the total number of reported diseases. Amongst the various skin diseases, rosacea deserves special attention [3]. It is a chronic disease affecting the skin and sometimes the eyes, manifested by redness, rashes and visible blood vessels. Rosacea accounts for ~0.5% of all skin diseases [4]. Zhangireyeva *et al*. [5] and Zubkova *et al*. [6] in 2023 focused on the fact that skin diseases can substantially reduce the quality of life of people, with the main harm being caused by a cosmetic defect. These diseases often lead to psychological problems such as low self-esteem and social isolation, as visible skin disorders can cause embarrassment and inconvenience in daily life and social interactions.

The relevance of examining this topic is due to the high prevalence of rosacea, the negative impact of the disease on the quality of life of patients and the complexity of therapy, especially in moderate and severe forms. Rosacea is characterized by pronounced cosmetic defects, which lead to psychological discomfort and social maladaptation in patients [7,8]. In addition, the lack of a single standard and optimal treatment algorithm makes it difficult to effectively manage patients with rosacea. According to Azizov *et al*. [9], one of the common theories explains the cause of rosacea by the presence of tiny Demodex mites that naturally live on human skin. Studies show that in patients with rosacea, the number of these mites can be substantially higher, which contributes to the development of inflammatory processes on the skin.

The problem of this study is the need to find new approaches to the treatment of moderate and severe forms of papulopustular rosacea, which would provide a faster and more pronounced clinical improvement compared with traditional methods. Despite the fact that various topical and systemic drugs for the treatment of rosacea are available in the arsenal of dermatologists, their effectiveness and safety in some cases remain insufficient [10,11]. Thus, Edilbekova *et al*. [12] highlight the products of natural origin as safe and affordable skin care products.

In recent years, increasingly more attention has been paid to the use of ivermectin, an antiparasitic agent with pronounced anti-inflammatory and immunomodulatory properties [13]. A number of studies have demonstrated the high effectiveness of topical ivermectin in the treatment of papulopustular rosacea, linking its mechanism of action with the elimination of Demodex mites and the suppression of inflammatory reactions. However, the potential of combination therapy using ivermectin in combination with systemic drugs (antibiotics, antiparasitic agents and antihistamines) in moderate and severe forms of rosacea has yet to be examined. The presented studies did not analyse the effect of antiparasitic drugs on the course and treatment of rosacea. These studies did not consider the potential therapeutic effects of such drugs on the symptoms and manifestations of this dermatological disease, which indicates a gap in the current understanding of the possibilities of an integrated approach to the treatment of rosacea using antiparasitic therapy.

The purpose of this cohort study is to evaluate the safety and effectiveness of combination therapy using 1% ivermectin solution in combination with systemic treatment methods in patients with moderate and severe forms of papulopustular rosacea. This goal is based on the need to find more effective approaches to the treatment of severe manifestations of rosacea, which would provide a substantial and rapid improvement in the clinical picture of the disease.

## Methods

This study was conducted on a sample of patients at the Osh Interregional Center for Dermatovenerology from December 2022 to April 2023. Seventy patients aged 29–64 years with moderate (*n*=39) and severe (*n*=31) forms of papulopustular rosacea were under observation.

The criteria for inclusion and exclusion of patients in this study were strictly defined to ensure reliable and representative results. Patients admitted to participate in the study had to meet the following inclusion criteria: age from 18 to 70 years and the established diagnosis of ‘papulopustular type of rosacea’. These parameters were chosen for the study to cover the adult population with the active phase of the disease, which would allow an adequate assessment of the effectiveness and safety of therapeutic interventions.

On the other hand, exclusion criteria were clearly defined to minimize risks and potential complications. The study did not include people over the age of 70 to avoid possible age-related complications and drug interactions that could affect the results of the study. Pregnant and breast-feeding women were also excluded due to the potential risk to the development of the foetus or newborn. Patients with other skin diseases, acute inflammatory processes on the skin, oncological diseases or allergic reactions in the anamnesis to the components of the examined drugs were also excluded to clearly determine the effect of rosacea treatment without cross-exposure to other diseases or conditions.

Most of the participants were women (*n*=63), while there were seven men. Notably, none of the women showed signs of pregnancy or lactation. The predominant skin phenotype, according to Fitzpatrick’s classification, corresponded to type 5; the study participants were representatives of the Kyrgyz and Uzbek ethnic groups living in the Batken region (*n*=31) as well as the Nookat (*n*=25) and Alai (*n*=14) regions of Kyrgyzstan. The study was conducted in compliance with ethical principles, and all patients provided written informed consent to participate. The patients were divided into two groups: the main group and the control group. The main group (*n*=70; 63 women and 7 men) received combination therapy, including 1% ivermectin solution, while the control group underwent traditional treatment (the use of trichopoly soap and Yam ointment).

The treatment of the main group was divided into two stages: inpatient (10–14 days) and outpatient (20–35 days); the total duration was 1–1.5 months. The therapy regimen included the following components:

1. Phase A (initial): conducting a clinical examination, laboratory tests of blood, urine, faeces and facial skin scraping to determine the presence of Demodex mites; the purpose of this stage was to exclude concomitant infectious diseases;
2. Phase B (stationary): systemic therapy, including taking doxycycline (100 mg two times a day for 10 days), intravenous metronidazole 0.5% (100 ml three times a day for 10 days), cetirizine (one time a day for 14 days) and enterosgel (1.5 tablespoons three times 2 h a day before meals);
3. Phase C (stationary): local therapy, consisting of two stages: C1 – applying a mixture of ichthyol solution and Vishnevsky liniment (1 : 1) to the affected areas of the facial skin under a bandage for 12 h (duration 3–5 days); C2 – applying 1% ivermectin solution to the facial skin with a gradual increase in exposure from 2 to 12 h daily for 7 days;
4. Phase D (outpatient): continued topical application of 1% ivermectin solution two times a day for 15 min for 20–35 days, the use of sunscreen (sun protection factor [SPF] 25–50) and adherence to a strict hypoallergenic diet for 3 months with a gradual easing of dietary restrictions.

The experimental design of the trial includes a stationary phase when ivermectin is administered after an initial inpatient treatment phase. This allows for a thorough assessment of topical and systemic medications in the management of moderate to severe rosacea. Before ivermectin is used topically, patients are stabilized during the inpatient period by systemic medications, such as doxycycline, intravenous metronidazole and cetirizine, which decrease inflammation and control bacterial and allergic reactions. Ivermectin’s introduction attempts to both preserve earlier advantages and target specific pathogenic processes linked to rosacea, namely the overabundance of Demodex mites that fuel inflammatory reactions.

This study’s evaluation criteria for rosacea used a well-recognized score system in dermatology to evaluate traditional, visually observed clinical symptoms, including erythema, telangiectasia, papules/pustules and oedema [14]. This approach is useful, but it can vary depending on the evaluator’s perspective and the surrounding circumstances. Although more objective measuring methods, such as laser Doppler imaging and digital image analysis, provide higher precision, their regular application is limited by their need for specialist tools and training. The main criterion for evaluating the effectiveness of treatment was the number of Demodex mites before and after therapy and the dynamics of clinical manifestations of rosacea, which was evaluated using a 4-point scale for erythema, telangiectasia, papules/pustules and oedema before treatment, at the second, fourth and eighth weeks of therapy.

## Results

Before starting treatment, a detailed assessment of the condition of the study participants was conducted in the main group, using a 4-point scale to determine the severity of each of the symptoms of rosacea. The initial average scores were for erythema – 2.7 with a standard deviation of 0.5; for telangiectasia – 2.3 with a standard deviation of 0.6; for papules/pustules – 2.6 with a standard deviation of 0.5; and for oedema – 2.4 with a standard deviation of 0.7. These values indicated moderate symptoms in the majority of the group members (Table 1).

**Table 1.** The scale of assessment of clinical manifestations of rosacea

Score	Erythema	Telangiectasia	Papule and pustule	Oedema
0	There is no noticeable erythema	Absent	Up to 10 elements	Absent
1	Mild erythema, limited either to the central area of the face or generalized	Mild severity: thin vessels 0.2 mm in diameter, occupying 10% of the face surface	11–20 elements	Weak
2	Moderate erythema, limited either to the central area of the face or generalized	Moderate severity: several thin vessels and/or several larger vessels with a diameter of more than 0.2 mm, occupying more than 0.2–30% of the face surface	21–30 elements	Moderate
3	Severe erythema or purple-red colour, either limited to the central area of the face or generalized	There are many small and/or large vessels occupying more than 30% of the face surface	More than 30 elements	Strong, progressive

Source: [14].

In the second week of therapy, there was already a slight decrease in the severity of symptoms in the main group. Erythema decreased to 2.1 points, which is a decrease of 22% from the baseline level. Telangiectasia decreased to 1.9 points, which represents a decrease of 17%. Papules and pustules decreased to 2 points, which corresponds to a decrease of 23%, while oedema decreased to 1.8 points, decreasing by 25%. These data indicate an initial positive effect of the therapy used on the condition of patients.

By the fourth week of treatment, a more noticeable improvement in the clinical picture was registered in the main group. The values of symptom reduction were expressed as follows: erythema decreased by 48%, reaching 1.4 points. Telangiectasia decreased by 43% to 1.3 points. Papules and pustules decreased by 54%, reaching 1.2 points. Oedema decreased by 54% to 1.1 points. These results confirm substantial progress and highlight the effectiveness of the therapeutic approach used. By the eighth week of therapy, an almost complete reduction of the main clinical symptoms was observed in the main group. Erythema decreased by 78% to 0.6 points, telangiectasia also decreased by 78% to 0.5 points, papules/pustules decreased by 85% to 0.4 points and oedema decreased by 88% to 0.3 points. This indicates the high effectiveness of therapy and highlights the long-term positive changes in the condition of patients.

In the control group receiving traditional treatment, the dynamics of clinical manifestations were substantially less pronounced. By the fourth week of treatment, the improvement ranged from 25 to 30%, depending on the type of symptom, and by the eighth week, there was a decrease in the severity of symptoms from 46 to 52%. These results, although showing some improvement, were less substantial compared with the main group.

For a more in-depth analysis of the influence of various factors on the dynamics of clinical symptoms, a regression analysis was performed, where changes in the severity of erythema, telangiectasia, papules/pustules and oedema by the fourth and eighth weeks of therapy were considered as dependent variables. Membership in the main or control group, age, gender and severity of the initial condition of the participants were included as independent variables.

The study identified dependent variables covering changes in the severity of key clinical symptoms such as erythema, telangiectasia, papules/pustules and oedema. These changes were measured and analysed at two key stages of treatment: at the fourth and eighth weeks of therapy. An assessment of the dynamics of these symptoms allowed assessing the progress of the participants and the effectiveness of the treatment methods used.

**Fig. 1.**
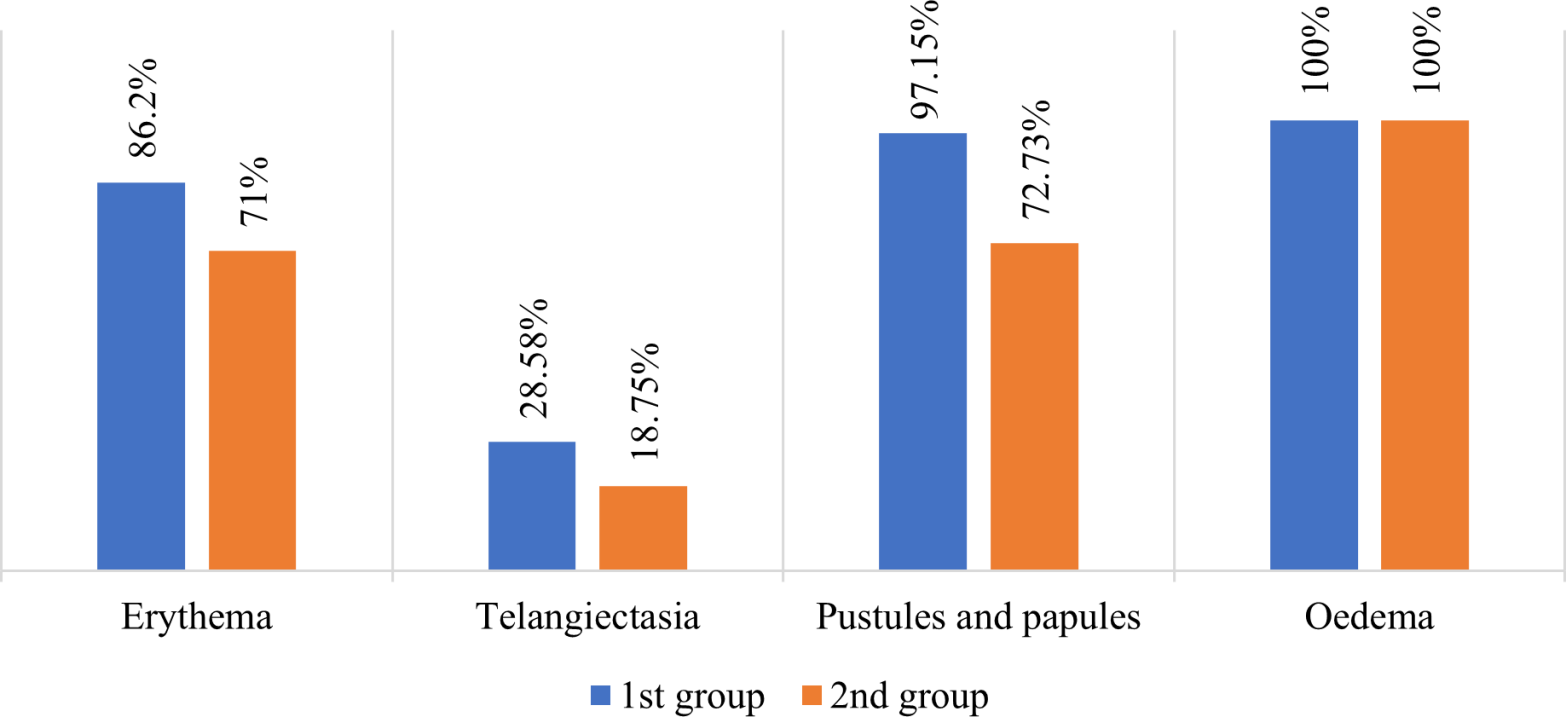
Effectiveness of new treatment protocol in comparison with established methods.

Several key parameters were included as independent variables in the analysis to determine the effect of various factors on treatment outcomes. First, the participants’ belonging to the main or control group was considered. The main group participated in combination therapy using ivermectin (new treatment protocol), while the control group followed the traditional course of treatment (established methods). This distinction clarified that the new treatment protocol involves a combined therapeutic approach incorporating ivermectin and systemic drugs, while the established methods refer to conventional therapies typically used in rosacea treatment. In addition, the analysis included the age of the participants, their gender and the severity of the initial health condition. These data helped the researchers understand how demographic and clinical characteristics can influence treatment response, which is important for optimizing treatment approaches and clarifying possible recommendations for different patient groups. The inclusion of these variables in the analysis was the key to understanding the complex effects of treatment on a diverse population of study participants (Fig. 1).

In this study, a multiple linear regression model was used to analyse and evaluate the relationship between various factors and changes in clinical symptoms in patients with rosacea by the fourth and eighth weeks of therapy [15]. According to this model, patient-specific variables, such as therapy type, demographics and baseline symptom intensity, affect how rosacea patients’ clinical symptoms develop. It measures how these factors affect treatment results using multivariate linear regression, acknowledging that personal traits like age and gender also matter. The model was expressed by the following equation:

(1) Y=β0 + β1Group + β2Age + β3Gender + β4Baseline_Severity + ε

where *Y* is a dependent variable, which means a change in the severity of one of the clinical symptoms (erythema, telangiectasia, papules/pustules and oedema) according to the results of these treatment periods; *β*0 is a constant or free term, which represents the basic value of the symptom change when all other independent variables are zero; *β*1 is the influence of belonging to the group (0 for the control group and 1 for the main group that received combination therapy with ivermectin) on the change in clinical symptom (this allows evaluating the effectiveness of combination therapy compared with traditional methods of treatment); *β*2 measures the change in clinical symptom for each year of increasing patient age, allowing analysis of how age affects the response to treatment; *β*3 represents differences in symptom change between men and women (0 for women and 1 for men), which helps to understand whether there are gender differences in the effectiveness of treatment; *β*4 is associated with the initial severity of the condition (Baseline_Severity), which shows how the initial severity of symptoms affects their subsequent change under the influence of therapy and *ε* reflects unpredictable factors that may affect the outcome and are not considered by other variables of the model (this may include individual responses to treatment, differences in the accuracy of measuring symptoms and other random variations).

Such a model allowed for the assessment of the extent to which group membership, age, gender and initial severity of the condition affect the dynamics of clinical symptoms of rosacea during treatment. Therewith, age, gender and initial severity of the condition did not substantially affect the dynamics of clinical symptoms. In addition, regression analysis confirmed that in the main group, there was a substantially more pronounced decrease in the number of Demodex ticks compared to the control group (regression coefficient −0.78, *P*<0.1).

**Fig. 2.**
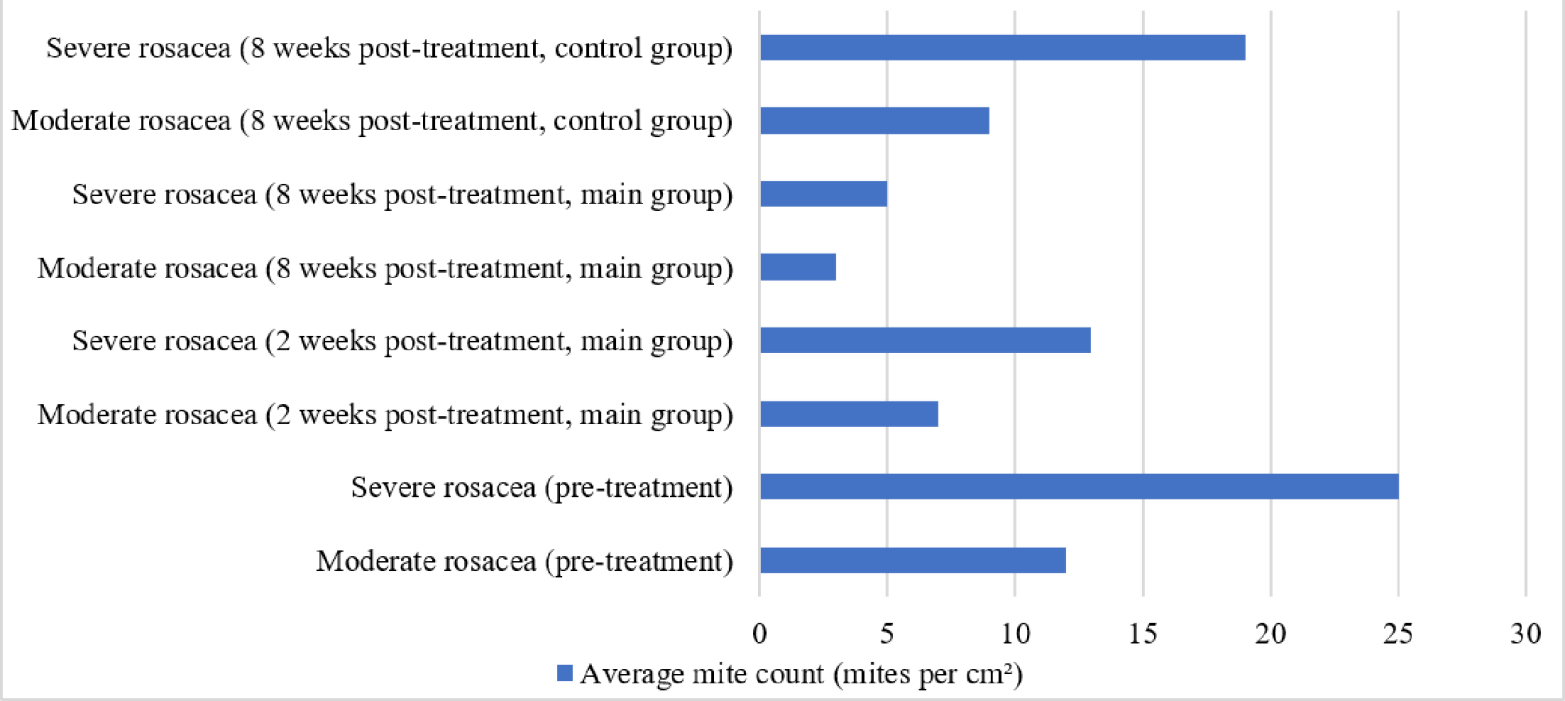
Effect of treatment on Demodex mite density in moderate and severe rosacea patients.

The primary method used for identifying Demodex mites was skin scraping, which entails carefully removing afflicted facial regions to obtain superficial skin samples. Individuals with mild papulopustular rosacea had an average of 10–15 mites per cm², whereas severe instances had 20–30 mites per cm², especially in regions with severe erythema and pustule development (Fig. 2). The severity of rosacea correlated with the mite counts, which varied greatly across individuals. Mite densities dropped by ~40% in moderate instances and 50% in severe cases following 2 weeks of combined therapy with 1% ivermectin and systemic medication. By the end of 8 weeks, counts had dropped to almost nothing, with 85% of patients having counts below 5 mites per cm^2^. Only slight decreases of 20–25% were observed in the control group that received conventional therapy, indicating the efficacy of ivermectin in addressing this root cause and confirming the theory that Demodex mites have a role in the pathophysiology of rosacea.

The results of the regression analysis confirmed the substantial effect of belonging to the main group receiving combination therapy with ivermectin on improving clinical manifestations in patients. This combination therapy was identified as the main predictor of a more pronounced improvement in clinical manifestations. In the fourth week of treatment, for example, the regression coefficient for erythema reached −0.64, which indicates that patients in the main group experienced a substantially greater decrease in the severity of erythema compared with the control group by 0.64 points.

Similar results were reported for other symptoms: regression coefficients for telangiectasias were −0.51 (*P*<0.1), for papules/pustules −0.7 (*P*<0.1) and for oedema −0.62 (*P*<0.1), which also indicates a substantial advantage of combination therapy in these cases. By the eighth week of therapy, the differences in improving clinical manifestations between the main and control groups became even more obvious. Regression coefficients for erythema increased to −0.91 (*P*<0.1), for telangiectasia to −0.86 (*P*<0.1), for papules/pustules to −1 (*P*<0.1) and for oedema up to −0.98 (*P*<0.1). These data confirm a much more substantial improvement in the clinical picture by the end of the follow-up period in patients of the main group.

The study conducted a detailed analysis of the influence of various factors, including age, gender and initial severity of the condition, on the dynamics of clinical symptoms in patients treated with rosacea. After careful statistical processing of the data, it was found that these variables do not have a statistically substantial effect on the change in clinical manifestations of the disease during treatment. Thus, the results confirmed that combination therapy using ivermectin is effective regardless of the age of patients, their gender or the severity of the disease at the initial stage. These findings are important for clinical practice, as they allow considering ivermectin as a universal therapeutic agent for a wide range of patients suffering from rosacea without the need for individual adaptation of the therapeutic approach considering demographic or clinical characteristics. This simplifies treatment protocols and expands the possibilities of using this method in different populations. In addition, it was established that in the main group, there was a substantially more pronounced decrease in the number of Demodex ticks, which further emphasizes the effectiveness of combination therapy compared with the control group. These data add weight to the conclusions about the benefits of using ivermectin as part of the complex therapy of skin diseases.

The regression coefficient for this indicator was −0.78 (*P*<0.1), indicating an almost 80% advantage of combination therapy with ivermectin in eliminating Demodex. At the same time, no serious side effects were reported in any patient receiving combination therapy with ivermectin. All patients tolerated the proposed treatment regimen well. Thus, the results of the regression analysis clearly demonstrate that the use of combination therapy using 1% ivermectin solution in combination with systemic drugs is a key factor determining faster and more pronounced clinical improvement in patients with moderate and severe papulopustular rosacea. This approach allows for achieving a substantial reduction in the severity of the main symptoms of the disease and effectively eliminating Demodex mites, which is an important pathogenetic component of rosacea.

The results of the study show that ivermectin has a role in improving symptoms on its own, since there was a notable reduction in the intensity of erythema, telangiectasia, papules and oedema following ivermectin administration. According to regression analysis, ivermectin significantly lowers clinical symptoms and mite counts, particularly after the fourth and eighth weeks of therapy, even if the first systemic treatment still lowers inflammation. This discovery lends credence to the idea that ivermectin has special therapeutic advantages that improve patient outcomes above and beyond those of systemic therapies alone. Therefore, rather than acting as a straightforward maintenance agent, the design and findings confirm that ivermectin plays a critical, active role in attaining prolonged improvement.

The results obtained indicate the high efficiency and safety of the proposed integrated approach to the treatment of this category of patients. In addition, regression analysis showed that the effectiveness of combination therapy with ivermectin does not depend on the age, gender and initial severity of the patients’ condition. However, it is advisable to evaluate the effect of combination therapy on the quality of life of patients and to investigate the long-term results of treatment. In particular, it is of interest to analyse how much the improvement in the clinical picture achieved during the study affects patients’ subjective perception of their skin condition and well-being. This is important because rosacea often negatively affects the psycho-emotional state of patients, reducing their quality of life. An assessment of this aspect could complement existing data on the effectiveness of combination therapy with ivermectin. In addition, a promising direction is to explore the possibility of maintaining the achieved improvement in the long term. Thus, it is important to understand how persistent the clinical response to the proposed treatment is and whether repeated courses of therapy are required to prevent relapses. Data on the long-term results of combination therapy with ivermectin could substantially expand the understanding of its potential in the treatment of rosacea.

In conclusion, the results of this study using regression analysis demonstrate that combination therapy using 1% ivermectin in combination with systemic drugs is a highly effective and safe method of treating moderate to severe papulopustular rosacea. This approach allows for faster and more pronounced clinical improvement compared with traditional therapies, regardless of the demographic characteristics of patients and the initial severity of their condition. The use of combination therapy with ivermectin can be a valuable addition to existing rosacea treatment strategies.

## Discussion

The results of the study demonstrate that the use of combination therapy using a 1% solution of ivermectin in combination with systemic drugs (antibiotics, antiparasitic agents and antihistamines) is an effective and safe method of treating moderate and severe forms of papulopustular rosacea.

One of the key observations was a more pronounced and rapid improvement in the clinical picture in the main group receiving combination therapy, compared with the control group, where traditional methods of treatment were used. By week 4, the study group showed a substantial decrease in the severity of erythema, telangiectasia, papules/pustules and oedema (by 48–54%), while in the control group, the improvement was only 25–30%. By the eighth week of treatment, an almost complete reduction of the main symptoms was observed in the main group (78–88% decrease), indicating the high effectiveness of the proposed combination therapy. Singh *et al*. [16] claimed that the pathogenesis of rosacea is due to an increase in the number of *Demodex folliculorum* mites in the skin, which leads to the activation of inflammation through Toll-like receptor-2. In this aspect, 1% ivermectin cream offers effective and specific treatment of moderate to severe rosacea due to its anti-inflammatory and acaricidal properties. A study by Schaller *et al*. [17] in 2022 established that the use of 1% ivermectin cream for 16 weeks effectively reduced not only the visible symptoms of rosacea, such as papules and pustules, but also invisible manifestations, including burning, dryness and itching. The cream substantially improved erythema and skin texture, which was confirmed by both clinical assessment and self-assessment of patients in the study by Schaller *et al*. This study also noted the effectiveness of this drug. Another retrospective study of 39 patients suffering from rosacea with persistent facial erythema and increased Demodex density revealed a substantial decrease in the assessment of carcinoembryonic antigen (CEA) and Demodex density after the use of topical ivermectin, both in isolation and in combination with oral carvedilol. Noticeable improvements were recorded in both groups (*P*<0.1), but substantial differences between the groups in the dynamics of changes in CEA (*P*=0.7 and *P*=0.23) and Demodex density (*P*=0.82 and *P*=0.1) were not detected [18], which coincides with the results of this study.

However, according to the results obtained by Logger *et al*. [19], in 20 patients, treatment with topical ivermectin led to a clinical decrease in inflammatory elements, but microscopy did not identify substantial changes in demodicosis, inflammation and vascular parameters. Notably, no serious side effects were reported in any patient receiving combination therapy with ivermectin. All patients tolerated the proposed treatment regimen well, which indicates its safety. A meta-analysis in 2023 examined various methods of controlling Demodex mites, including ivermectin, tea tree oil (TTO) and permethrin. During the first month of treatment, the effect size (ES) ranged from 0.7 (cleanser) to 1.95 (systemic ivermectin-metronidazole). In the second month, ES increased to 4.4 (topical ivermectin), and in the third month, it reached 8.37 (topical ivermectin), showing the high effectiveness of topical ivermectin in reducing the Demodex tick population. The rate of decrease in the number of ticks in topical ivermectin, TTO, permethrin and intense pulsed light is close to 100%. The side effects were mostly mild, with no reports of serious side effects, which highlights the safety of using these methods [20]. There were also no serious side effects in this study, which indicates the safety of ivermectin. The purpose of the study was to evaluate the efficacy and safety of various prescriptions and doses of antibiotics in the treatment of rosacea using a network meta-analysis. According to the analysis of 31 randomized trials with 8226 patients, ivermectin showed the best efficacy in the treatment of papulopustular rosacea with a low risk of adverse events. There were also no side effects [21]. This study also confirms the safety of ivermectin.

The results of the regression analysis confirmed that belonging to the main group receiving combination therapy with ivermectin was a key predictor of a more substantial improvement in the clinical manifestations of rosacea. Thus, regression coefficients for assessing the dynamics of erythema, telangiectasia, papules/pustules and oedema at weeks 4 and 8 were substantially higher in the main group compared with the control group. This indicates that combination therapy with ivermectin provided a much more pronounced reduction in the severity of the main symptoms of the disease. In the clinical trial by Yeh *et al*. [22] in 2022, with the participation of 33 patients, of whom 27 completed the study, it was shown that both ivermectin and metronidazole substantially improve facial erythema in patients with rosacea after a month of treatment. However, there were no substantial differences between the two drugs over a 3-month period in terms of overall erythema reduction. Subjective evaluations indicated that ivermectin was more effective than metronidazole in improving symptoms of erythema and warmth after 2 months, and itching and roughness after 3 months. Despite these results, after 3 months of treatment, there was no difference in overall improvement between the two treatments, which highlights the need for individual treatment plans based on specific symptoms and patient reactions. In this study, the main progress was also noted in the first 4 weeks after the start of treatment. A study by Osman *et al*. [23] in 2022 showed that pulsed red laser (PDL) is effective in the treatment of rosacea, and the addition of a cream with 1% ivermectin can enhance this effectiveness. During the experiment, patients were treated only with PDL or in combination with ivermectin, while no serious side effects were observed, which confirms the safety of the methods used. Notably, the visible effect also occurred after 4 weeks of therapy, as in the present study.

The effectiveness of combination therapy did not depend on the age, gender and initial severity of the patients’ condition. Regression analysis did not identify a substantial influence of these factors on the dynamics of clinical manifestations, which emphasizes the universality of this therapeutic approach. A paper by Ávila *et al*. [24] in 2021 showed high efficacy and safety of the combined gel ivermectin (0.1%) and metronidazole (1%) in the treatment of Demodex-associated blepharitis with complete destruction of ticks in 96.6% of patients and no side effects. In this study, ivermectin monotherapy was used, and it was highly effective. In addition, the results of the study demonstrated that in the main group receiving combination therapy with ivermectin, there was a substantially more pronounced decrease in the number of Demodex mites compared with the control group [25]. This fact is consistent with the mechanism of action of ivermectin aimed at the elimination of these parasites, which are considered one of the important pathogenetic factors in the development of rosacea [26]. The study analysed the effectiveness of topical ivermectin as an anti-inflammatory and antidemodectic agent in the treatment of papulopustular rosacea, with special attention to the periods between relapses. During the prospective follow-up of patients who achieved complete recovery, the time to the first relapse and the frequency of relapses of infection with Demodex mite and rosacea were evaluated. The success rate of treatment from Demodex infestation was 87.5%, while relapses were reported in 12.5% of patients. The average time to relapse was 152 days. Based on the data, a scheme of supportive treatment with ivermectin twice a week is proposed to minimize relapses [27]. Similar results were obtained in this study.

The results obtained are consistent with data from a number of other studies indicating the high efficacy and favourable safety profile of ivermectin in the treatment of papulopustular rosacea. The use of ivermectin in combination with systemic drugs, as shown in this study, allows for a faster and more pronounced improvement in the clinical picture compared with traditional approaches. A study by Sobolewska *et al*. [28] evaluated the effectiveness of using a cream with 1% ivermectin in the treatment of rosacea of the eyes and skin. The results of a pilot study on ten patients showed substantial improvement in the condition of the eyes and skin, including a decrease in blepharitis and conjunctival redness and an improvement in the general condition of the cornea. These positive effects were observed already by the 16th week and persisted until the end of the follow-up for 8 months, without noted undesirable effects, confirming the safety and effectiveness of daily use of ivermectin cream for the treatment of rosacea.

The combination of the antiparasitic, anti-inflammatory and immunomodulatory effects of ivermectin, in addition to systemic therapy, provides a comprehensive effect on the key pathogenetic mechanisms of rosacea. Effective elimination of Demodex mites, along with a decrease in the severity of the main symptoms, is an important factor determining the high effectiveness of combination therapy. Based on the results of a retrospective review of Huang *et al*. [18], 39 patients with rosacea, the use of topical ivermectin in combination with oral carvedilol led to a substantial decrease in the clinical assessment of CEA and demodicosis density (both *P*<01). Three patients from the ivermectin-only group refused treatment due to an early exacerbation of rosacea caused by ivermectin. In the remaining groups (patients receiving only ivermectin, *n*=14; ivermectin-carvedilol, *n*=22), there was no statistically substantial difference in CEA before and after treatment (*P*=7 and *P*=23, respectively) and in demodicosis density (*P*=82 and *P*=10, respectively). Of the 36 patients, the response was excellent in 26 (72 %), good in 2, satisfactory in 4 and none in 4. In this study, a good result was noted in the mono mode, but the combined treatment with this drug was not examined.

However, for a more complete assessment of the potential of the proposed therapeutic approach, further studies with a larger sample size and a longer follow-up period are needed. It is also of interest to investigate the effect of combination therapy with ivermectin on the quality of life of patients and long-term treatment results.

## Conclusions

The results of the cohort study demonstrate the high efficacy and safety of combination therapy using 1% ivermectin solution in combination with systemic drugs (antibiotics, antiparasitic agents and antihistamines) for the treatment of moderate and severe forms of papulopustular rosacea. An analysis of the dynamics of clinical manifestations showed that in the main group receiving combination therapy with ivermectin, there was a faster and more pronounced improvement compared with the control group, where traditional methods of treatment were used. By week 4, a substantial decrease in erythema, telangiectasia, papules/pustules and oedema was achieved in the main group (by 48–54%), while in the control group, the improvement was only 25–30%. By the eighth week of therapy, an almost complete reduction of the main symptoms was observed in the main group (78–88% decrease).

The results of the regression analysis confirmed that the belonging of patients to the main group receiving combination therapy with ivermectin was a key predictor of a more substantial clinical improvement, regardless of age, gender and initial severity of the condition. In addition, a much more pronounced decrease in the number of Demodex mites was recorded in the main group, which is consistent with the mechanism of action of ivermectin. Notably, no serious side effects were reported in any patient receiving combination therapy with ivermectin, which indicates that the proposed therapeutic approach is well tolerated.

Thus, the results of this study indicate the high efficacy and safety of combination therapy using 1% ivermectin solution in the treatment of moderate and severe forms of papulopustular rosacea. This approach can be a valuable addition to existing strategies for the treatment of this chronic dermatological disease. Studies with longer follow-up periods and an assessment of their impact on the quality of life of patients are needed to further explore the potential of combination therapy with ivermectin.

## Abbreviations

CEA: carcinoembryonic antigen
ES: effect size
IPL: intense pulsed light
PDL: pulsed red laser
TTO: tea tree oil

## References

[1] Parraga SP, Feldman SR (2023). Biomarkers in rosacea: a systematic review. J Eur Acad Derm Venereol.

[2] Mukhanova G, Ospanaliyeva M, Kamaliyeva M, Duisenbayeva B, Kenzhekulova R (2022). Incidence of morbidity among children and adolescents in Kazakhstan. J Health Develop.

[3] Polatova DSh, Ibragimova DА, Madaminov АY, Davletov RR, Savkin АV (2023). Dynamics of the incidence of non-melanoma malignant neoplasms of the skin in the republic of Uzbekistan for the 2018–2022. Sark Kost MagTkan Opuh Koz.

[4] Ospanov E, Kanatbayev S, Akshalova P, Mamanova S, Bashenova E (2023). Risks of emergence and spread of nodular skin disease virus in the republic of kazakhstan. Ġyl Ža̋ne Bìlìm.

[5] Zhangireyeva AZ, Aliyeva RK, Iztleuova GM, Balmanova ZM, Seitbaeva VA (2023). SCORAD index patients’ application during atopic dermatitis as control and prevention of disease aggression. West Kazakh Med J.

[6] Zubkova L, Bocharov V, Bocharova V, Filatova L (2022). Mechanisms of disorders of psychophysiologic state of women with rosacea. Sci Rev.

[7] Ravshanov AX, Suhail M, Komilova N, Ravshanov S (2024). Medical geographical zoning in part of Uzbekistan – A regional synthesis. Reg Sci Pol Pract.

[8] Hartmane I (2024). Study of genetic mutations and their association with the development of atopic dermatitis and other skin diseases. Plast Aesth Nurs.

[9] Azizov BS, Nurmatova IB, Ayupova ST (2023). Improving the methods of rational therapy for demodicosis. Adv Ophthalmol.

[10] Rovira RH, Tuzhanskyy SY, Pavlov SV, Savenkov SN, Kolomiets IS (2016). Polarimetric characterisation of histological section of skin with pathological changes. Proceed SPIE Int Soc Optic Eng.

[11] Rubins AY, Hartmane IV, Lielbriedis YM, Schwartz RA (1992). Therapeutic options for erythroderma. Cut.

[12] Edilbekova A, Isakova K, Razzakov A (2022). Trendy remedies with natural origin used in the cosmetology. Bull Sci Pract.

[13] Lutsak I, Litvinenko D, Meleha K, Barabanchyk O, Savchuk A (2022). Rational pharmacotherapy of respiratory diseases in the COVID-19 pandemic. Int J pharm res allied sci.

[14] Wienholtz NKF, Thyssen JP, Christensen CE, Thomsen SF, Karmisholt KE (2023). Validity and reliability of the rosacea area and severity index: a novel scoring system for clinical assessment of rosacea severity. J Eur Acad Dermatol Venereol.

[15] Naik PP (2021). Emerging treatment options for rosacea. Curr Derm Rep.

[16] Singh R, Perche PO, Kelly KA, Cook MK, Balogh EA (2023). Topical ivermectin is associated with improved erythematotelangiectatic, papulopustular, and phymatous rosacea in a secondary analysis. J Drugs Dermatol.

[17] Schaller M, Riel S, Bashur R, Kurup N, Schnidar H (2022). Ivermectin treatment in rosacea: how novel smartphone technology can support monitoring rosacea-associated signs and symptoms. Dermatol Ther.

[18] Huang H, Hsu C, Lee J (2021). Rosacea with persistent facial erythema and high *Demodex* density effectively treated with topical ivermectin alone or combined with oral carvedilol. Dermatol Ther.

[19] Logger JGM, Peppelman M, van Erp PEJ, de Jong EMGJ, Nguyen KP (2022). Value of reflectance confocal microscopy for the monitoring of rosacea during treatment with topical ivermectin. J Dermatol Treatm.

[20] Li J, Wei E, Reisinger A, French LE, Clanner-Engelshofen BM (2023). Comparison of different anti-demodex strategies: a systematic review and meta-analysis. Dermatology.

[21] Xiao W, Chen M, Wang B, Huang Y, Zhao Z (2023). Efficacy and safety of antibiotic agents in the treatment of rosacea: a systemic network meta-analysis. Front Pharmacol.

[22] Yeh MCH, Tsai J, Huang YC, Wang H-H (2022). Topical metronidazole versus ivermectin for low-density *Demodex* rosacea: a rater-blinded, randomized, split-face trial. Acta Derm Venereol.

[23] Osman M, Shokeir HA, Hassan AM, Atef Khalifa M (2022). Pulsed dye laser alone versus its combination with topical ivermectin 1% in the treatment of rosacea: a randomized comparative study. J Dermatol Treatm.

[24] Ávila MY, Martínez-Pulgarín DF, Rizo Madrid C (2021). Topical ivermectin-metronidazole gel therapy in the treatment of blepharitis caused by *Demodex* spp.: a randomized clinical trial. Contact Lens & Anterior eye. J Brit Contact Lens Assoc.

[25] Hashizume H, Ishikawa Y, Hata A (2024). Increased *Demodex* mites after dupilumab therapy in facial skin: a case report. J Dermatol.

[26] Sharma A, Kroumpouzos G, Kassir M, Galadari H, Goren A (2022). Rosacea management: a comprehensive review. J Cosmet Dermatol.

[27] Trave I, Micalizzi C, Cozzani E, Gasparini G, Parodi A (2022). Papulopustular rosacea treated with ivermectin 1% cream: remission of the *Demodex* mite infestation over the time and evaluation of clinical relapses. Dermatol Pract Concept.

[28] Sobolewska B, Doycheva D, Deuter CM, Schaller M, Zierhut M (2021). Efficacy of topical ivermectin for the treatment of cutaneous and ocular rosacea. Ocular Immunol Inflamm.

